# Pan-Echinocandin-Resistant *Candida glabrata* Bloodstream Infection Complicating COVID-19: A Fatal Case Report

**DOI:** 10.3390/jof6030163

**Published:** 2020-09-06

**Authors:** Brunella Posteraro, Riccardo Torelli, Antonietta Vella, Paolo Maria Leone, Giulia De Angelis, Elena De Carolis, Giulio Ventura, Maurizio Sanguinetti, Massimo Fantoni

**Affiliations:** 1 Dipartimento di Scienze Biotecnologiche di Base, Cliniche Intensivologiche e Perioperatorie, Università Cattolica del Sacro Cuore, 00168 Rome, Italy; brunella.posteraro@unicatt.it (B.P.); giulia.deangelis78@gmail.com (G.D.A.); 2 Dipartimento di Scienze Gastroenterologiche, Endocrino-Metaboliche e Nefro-Urologiche, Fondazione Policlinico Universitario A. Gemelli IRCCS, 00168 Rome, Italy; paolomaria.leone@policlinicogemelli.it; 3 Dipartimento di Scienze di Laboratorio e Infettivologiche, Fondazione Policlinico Universitario A. Gemelli IRCCS, 00168 Rome, Italy; riccardo.torelli@policlinicogemelli.it (R.T.); antonietta.vella@policlinicogemelli.it (A.V.); elena.decarolis@policlinicogemelli.it (E.D.C.); giulio.ventura@unicatt.it (G.V.); massimo.fantoni@unicatt.it (M.F.); 4 Dipartimento di Sicurezza e Bioetica, Università Cattolica del Sacro Cuore, 00168 Rome, Italy

**Keywords:** SARS-CoV-2, coinfection, diabetes, bloodstream infection, *Candida glabrata*, echinocandin resistance, *FKS* mutation

## Abstract

Coinfections with bacteria or fungi may be a frequent complication of COVID-19, but coinfections with *Candida* species in COVID-19 patients remain rare. We report the 53-day clinical course of a complicated type-2 diabetes patient diagnosed with COVID-19, who developed bloodstream infections initially due to methicillin-resistant *Staphylococcus aureus*, secondly due to multidrug-resistant Gram-negative bacteria, and lastly due to a possibly fatal *Candida glabrata*. The development of *FKS*-associated pan-echinocandin resistance in the *C. glabrata* isolated from the patient after 13 days of caspofungin treatment aggravated the situation. The patient died of septic shock shortly before the prospect of receiving potentially effective antifungal therapy. This case emphasizes the importance of early diagnosis and monitoring for antimicrobial drug-resistant coinfections to reduce their unfavorable outcomes in COVID-19 patients.

## 1. Introduction

Since the beginning of the respiratory tract infection epidemic in China [1] caused by the 2019 severe acute respiratory syndrome coronavirus 2 (SARS-CoV-2), known as coronavirus disease 2019 (COVID-19), a substantial number of COVID-19 associated deaths have been reported worldwide [2]. While sepsis may be a fatal complication of COVID-19 [3], coinfection (also named superinfection) with bacteria or fungi may occur, albeit confined to the respiratory tract [4,5]. In two independent studies from Chinese hospitals, 27 (96.4%) of 28 [6] and 11 (16%) of 68 [7] COVID-19 patients who died had secondary infections. This is consistent with failed homeostasis between innate and adaptive responses [8] or a pronounced immune suppression [9], which is partly dependent on the loss of lymphocytes, following SARS-CoV-2 infection [10]. Diabetes is the most common comorbidity in COVID-19, with its late complications (e.g., ischemic heart disease) contributing to further increases in COVID-19 severity [11]. Additionally, diabetes increases not only the risk of infections [11] but also that of infection-related deaths [12]. In this context, diabetes seems to alter the intestinal barrier function, allowing gut microbiota members (e.g., *Enterobacterales* or *Candida* species) to reach the bloodstream and then to spread systemically [13].

Unlike invasive pulmonary aspergillosis, which has emerged as a secondary disease in COVID-19 patients with acute respiratory distress syndrome (ARDS) [14], invasive fungal diseases such as candidiasis and/or candidemia seem to be underestimated in the context of COVID-19. This is surprising, particularly when thinking of *Candida glabrata* [15], a common fungal commensal living on mucosal surfaces, which is the second leading cause of bloodstream infection (candidemia) in some countries, including the USA, Asia and European countries [16,17]. Among *Candida* species displaying multidrug resistance (e.g., co-resistance to azoles and echinocandins), *C. glabrata* is also known for its high tolerance to antifungal drugs [15]. Additionally, as this species has a tropism that causes candidemia among the elderly, COVID-19 patients suffering from ARDS (who are mostly elderly) could be prone to developing candidemia due to *C. glabrata*. This will be of particular concern in the case of COVID-19 patients with candidemia caused by echinocandin-resistant *C. glabrata*, because this species is intrinsically less azole susceptible, and consequently, the use of polyene antifungal drugs (i.e., amphotericin B) due to renal toxicity is largely limited among the elderly. It is noteworthy that COVID-19 itself is associated with kidney injury, which may further hamper the utility of amphotericin B in this context.

We describe the case of a COVID-19 patient with complicated type-2 diabetes who developed a bloodstream infection due to a *Candida glabrata* isolate that acquired pan-echinocandin resistance after 13 days of caspofungin treatment. The patient died of septic shock in the intensive care unit (ICU), shortly before the prospect of receiving potentially effective antifungal therapy.

## 2. Case Report and Results

A 79-year-old male presented to the emergency department in April 2020 with cough and dyspnea, following a suspected COVID-19 diagnosis because of his previous contact with a SARS-CoV-2 positive patient in a rehabilitation facility. Two days prior to admission (defined as day 1), he had been suffering from fever (38.0 °C). His 6-year medical history was significant for poorly controlled type-2 diabetes, ischemic heart disease and a stadium IV (necrosis and/or gangrene of the limb) peripheral artery disease treated with lower extremity revascularization, which culminated in left leg amputation in 2019. On physical examination, the amputated leg stump displayed necrotic and ulcerative lesions, whereas the patient was afebrile and negative for abnormal lung sounds and had a 98% blood oxygenation. His leucocytes (× 10^9^/L) were normal (4.7; normal range 4.0–10.0), whereas his serum creatinine (mg/dL) (1.3; normal range 0.7–1.2), C-reactive protein (CRP, mg/L) (37.8; normal range 0.0–5.0) and interleukin 6 (IL6, ng/L) (13.6; normal range 0.0–4.4) were altered. The patient’s chest X-ray and computed tomography findings were consistent with pneumonia, and positive SARS-CoV-2 RNA detection results (*C_T_* 30.3; E gene [18]) on nasal/pharyngeal swabs obtained in the emergency department allowed confirmation of the COVID-19 diagnosis [19]. Subsequent nasal/pharyngeal swabs taken from the patient at different times from admission tested positive for SARS-CoV-2 RNA.

The patient was transferred to the COVID-19 care unit, where he was started on antiviral therapy (which was continued for the next five days) with darunavir/ritonavir (800/100 mg q24h) combined with hydroxychloroquine (200 mg q12h), which was our national policy at that time. On days 4 and 5, the patient’s clinical conditions worsened, and his serum creatinine, CRP and leukocytes increased to 3.5 mg/dL, 155.4 mg/L and 6.9 × 10^9^/L, respectively. The patient developed fever (38.2 °C), a productive cough, and his blood oxygenation decreased to 92%, demanding oxygen administration through a Venturi mask (fraction of inspired oxygen, 24%). Due to highly suspected bacterial superinfection, he received empirical treatment with piperacillin/tazobactam (2.25 g q6h).

On day 8, the patient was still febrile (38.5 °C), his serum creatinine (3.9 mg/dL), CRP (177.2 mg/L) and leukocytes (9.4 × 10^9^/L) rose further, and his blood cultures from day 5 grew a methicillin-resistant *Staphylococcus aureus* organism. Consequently, piperacillin/tazobactam was discontinued and teicoplanin (200 mg q24h) was started. He improved, and subsequent blood cultures, a transthoracic echocardiogram and ultrasound studies to evaluate deep vein thrombosis were all negative. On day 25, teicoplanin was discontinued. The next day, both orthopedic and vascular surgeons who evaluated the patient decided on a new, more proximal amputation of his left leg. On day 27, the patient became febrile (38.5 °C). His leukocytes increased to 10.8 × 10^9^/L and infection indexes, including procalcitonin (PCT; normal range, 0.0–0.5 ng/mL), were elevated (CRP, 275 mg/L; PCT, 1.65 ng/mL). While his kidney injury seemed to recover (serum creatinine, 1.5 mg/dL), the patient became stably anemic (hemoglobin, g/dL; 7.4; normal range 13.0–17.0), requiring regular blood transfusions (until two days before death). On day 28, blood cultures from day 27 grew *Morganella morganii* (found to be resistant to cephalosporins and piperacillin/tazobactam but susceptible to carbapenems), which prompted initiation of antibiotic therapy with ertapenem (1 g q24h). Concomitantly, cultures from a progressively enlarging ulcer on the patient’s leg stump revealed growth of *Proteus mirabilis*, *Klebsiella pneumoniae* and *Escherichia coli* (all found to be susceptible to carbapenems).

On day 35, the patient again became febrile (38.2 °C) but CRP decreased (177.2 mg/L) and leukocytes remained unchanged (9.3 × 10^9^/L). Blood cultures yielded a yeast organism, later identified as *C. glabrata* using a previously described matrix-assisted laser desorption/ionization time-of-flight (MALDI-TOF) mass spectrometry-based method [20]. The isolate (defined as isolate 1) was susceptible to anidulafungin, micafungin and caspofungin, with MICs of 0.03, 0.03 and 0.06 μg/mL (SensititreYeastOne^®^ method; Thermo Fisher Scientific, Cleveland, OH, USA), according to the Clinical and Laboratory Standards (CLSI) clinical breakpoints [21]. On day 37, the patient started to take caspofungin (70 mg loading dose, day 1; 50 mg q24h, subsequent days). Blood cultures from day 39 were negative. After 13 days of antifungal therapy, the patient became febrile again (38.3 °C), and his blood parameters (creatinine, 2.71 mg/dL; leukocytes, 12.48 × 10^9^/L) and infection indexes (CRP, 278.4 mg/L; PCT, 20.58 ng/mL) were abnormal. On day 49, blood cultures were positive for *Acinetobacter baumannii* (found to be only susceptible to colistin) and again for *C. glabrata*. While ertapenem was discontinued and colistin (2.25 mUI q12h) was started, the patient continued to receive caspofungin. Shortly after (day 51), antifungal susceptibility testing was repeated on two morphologically different *C. glabrata* isolates that grew from blood cultures. One of the isolates (defined as isolate 2) revealed increased MICs of anidulafungin, micafungin and caspofungin, indicating resistance to all echinocandins (as discussed below).

On day 52, the patient underwent surgery for the previously planned left leg re-amputation. Unfortunately, on the same day of surgery and before the patient could eventually benefit from antifungal therapy change (i.e., amphotericin B instead of caspofungin) based on available antifungal susceptibility results, his clinical conditions worsened. The patient was immediately transferred to the ICU due to refractory septic shock, as identified by the receipt of vasopressor therapy and the elevated lactate (mEq/L) level (4.2; normal range 0.0–2.0) despite adequate fluid resuscitation. On day 53, the patient died.

**Figure 1:**
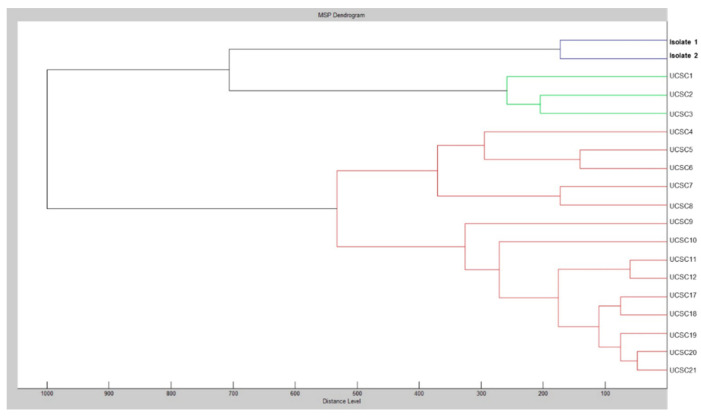
Cluster analysis of matrix-assisted laser desorption/ionization time-of-flight (MALDI-TOF) mass spectra obtained for 19 *C. glabrata* isolates, including the patients’ isolates 1 and 2. Shown is a dendrogram in which the distance between isolates is indicated as relative units. Zero means complete similarity and 1000 means complete dissimilarity. An arbitrary distance level of 500 was chosen to assess clustering among isolates.

Table 1 summarizes the results of both antifungal susceptibility testing and *FKS2* gene sequencing for *C. glabrata* isolates 1 and 2. Only for echinocandin antifungal agents, MIC values obtained with the SensititreYeastOne^®^ method were confirmed by the CLSI M27-A3 reference method [21]. As noted, except for all three echinocandins, the antifungal susceptibility profile of isolate 2 did not change compared to that of isolate 1. According to the echinocandin-resistant breakpoint values established by the CLSI [18], isolate 2 showed resistance to anidulafungin (MIC, 2 mg/L), caspofungin (MIC, 8 mg/L) and micafungin (MIC, 8 mg/L). Conversely, isolate 1 had echinocandin MICs (anidulafungin and micafungin, 0.03 mg/L; caspofungin, 0.06 mg/L) below the CLSI echinocandin-resistant breakpoint values [22]. Interestingly, both the isolates showed an intermediate susceptibility to fluconazole (MIC, 8 mg/L) and, according to the epidemiological cutoff values established by the CLSI [23], a wild-type susceptibility to amphotericin B, and the other azole (itraconazole, posaconazole and voriconazole) antifungal agents tested. A sequence analysis of the *FKS1*/*FKS2* genes [24] allowed us to identify T1976A (hot spot 1) and A3997T (hot spot 2) mutations in the *FKS2* gene, which resulted in an F659Y or I1333F amino acid change, respectively, with the former being already known [16,25,26] and the latter probably responsible for the observed echinocandin resistance. Furthermore, the MALDI-TOF MS-based analysis of profiles from *C. glabrata* isolates 1 and 2 allowed for comparing them with each other and with profiles from a clinical collection of *C. glabrata* isolates, which had been cultured from sterile or mucosal site samples (UCSC1–12, UCSC17–21). In particular, using the Bruker Daltonics BioTyper 3.0 software, raw spectra from the isolates were matched (with default parameter settings) against the main spectra from an in-house database [20]. Then, the integrated statistical tool Matlab 7.1 of the Biotyper 3.0 software allowed for generating a dendrogram (representation of hierarchical cluster analysis) of spectra to obtain graphical distance values between the isolates. As shown in Figure 1, the dendrogram resulting from the MALDI-TOF MS cluster analysis strongly suggested identity for *C. glabrata* isolates 1 and 2. It is likely that next-generation sequencing analysis could have provided greater discrimination/evidence of similarity among the isolates studied. However, a multilocus sequence-typing scheme (https://pubmlst.org/cglabrata/) showed that isolate 1 was the parental isolate from which originated isolate 2. Indeed, both the isolates shared the sequence type 22 for the analyzed loci *FKS*, *LEU2*, *NMT1*, *TRP1*, *UGP1* and *URA3* (7-5-6-12-1-8).

## 3. Discussion

**Figure 2:**
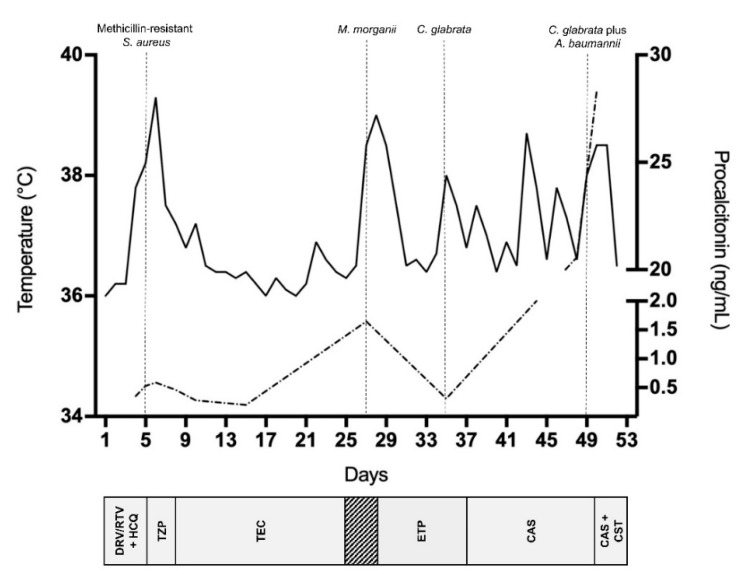
Timeline of major microbiological events during the patient’s clinical course and relative antimicrobial treatments. Fever (solid line) or procalcitonin (dashed line) patterns are shown. DRV/RTV, darunavir/ritonavir; HCQ, hydroxychloroquine; TZP; piperacillin/tazobactam; TEC, teicoplanin; ETP, ertapenem; CAS, caspofungin; CST, colistin.

This case illustrates the 53-day clinical course of a COVID-19 patient with persistent SARS-CoV-2 infection (repeated nasal/pharyngeal swabs tested positive for SARS-CoV-2 RNA) who needed protracted hospitalization, probably attributed to his major comorbidity (diabetes with its vascular complications). The patient met the clinical (fever, cough and dyspnea), laboratory (high CRP) and imaging (unilateral pneumonia) features recently recognized as COVID-19 hallmarks [10]. Yet, this case emphasizes the current uncertainty about the clinical disease evolution, partly linked to the presence of risk factors for either admission to the ICU or a fatal outcome of hospitalized patients [10]. In our patient, a succession of bloodstream infections, initially due to methicillin-resistant *S. aureus*, secondly due to multidrug-resistant Gram-negative bacteria, and lastly due to a possibly fatal echinocandin-resistant *C. glabrata*, outlined the COVID-19 associated clinical course (Figure 2).

At least three relevant causes might have contributed to determining fatal illness in the present case. First, COVID-19, which has significantly been associated with complications and deaths [10]. Second, type-2 diabetes, which remains a major comorbidity for severe COVID-19 [10,27] and increases the risk of mortality, especially in individuals with poorly controlled blood glucose [28]. Third, superinfection, which represents a new albeit scarcely studied condition in COVID-19 [5], particularly for invasive fungal infections [14,29]. The peculiar pathophysiology of either diabetes [11] or COVID-19 [30] may account for the occurrence of bacterial and fungal coinfections in our case, as in other cases [3,31]. The diabetes-induced immune dysregulation may exacerbate the virus-activated hyper-inflammatory “cytokine storm”, which in turn leads to complications (e.g., ARDS, shock, multiorgan failure and death) seen in severe COVID-19 phases [10]. However, diabetes (or other comorbidity) and COVID-19 commonly coexist during patients’ hospital stay as risk factors for fungal infection [29], although the extensive use of antibiotics and multiple bacteremias (as in this case) significantly predisposes one to development of candidemia. If candidemia was the immediate cause of death in our patient, it remains a matter of debate considering that the death was preceded by a surgical intervention, which may be relevant to the patient’s outcome.

In our patient’s disease phase upon his admission to the hospital, COVID-19 together with diabetes might have created a milieu that allowed microorganisms (e.g., *C. glabrata*, the last in the temporal sequence), including those resistant to antimicrobial agents, to thrive (likely in the gastrointestinal tract) and, hence, reach the bloodstream [32,33]. Immunosuppression and mucosal barrier disruption are, among others, well-recognized factors for isolation of *C. glabrata* from patient blood cultures [34] and, to some extent, bloodstream isolates are in vitro resistant to echinocandins [16,25,35]. This poses a great challenge for patient management [36] because echinocandins represent the first line of treatment in cases of invasive *C. glabrata* infections, including candidemia [37], due to the intrinsic low level of *C. glabrata* susceptibility to azoles (which was not the case of our patient’s isolates) [22].

Ultimately, the appearance of echinocandin resistance in our patient’s *C. glabrata* isolate aggravated the feared adverse prognosis of candidemia [38]. We provided the evidence of an in vivo development of *FKS*-associated echinocandin resistance during the patient’s treatment with caspofungin, consistent with previous case reports [26,39,40]. In two of them, echinocandin-resistant isolates were recovered from blood cultures of patients who had recurrent or persistent *C. glabrata* infections, thus implying micafungin treatments for 86 days in one case [26] and 30 days in the other case [39]. In another one [40], echinocandin resistance emerged within 8 days of the patient’s treatment with micafungin, and surprisingly, the patient had no previous or prolonged echinocandin exposure [41], but only uncontrolled diabetes, as a potential risk factor for microbiological failure. The abdominal cavity and mucosal surfaces are reservoirs for *Candida* species and a potential source for antifungal resistance due to uneven drug penetration [42,43]. Considering *C. glabrata*’s high propensity for acquiring in vitro resistance following echinocandin exposure [44], it is possible that an underlying gastrointestinal disorder or dysbiosis acted as selectors of *FKS* mutant *C. glabrata* subpopulations in our, as in other [40], case patients. Notably, a study assessing the emergence of in vitro resistance for the three echinocandins showed that 82 of 247 *C. glabrata* breakthrough isolates (i.e., bloodstream isolates exposed to each echinocandin agent) harbored *FKS* hot spot mutations, of which 6 were in *FKS1* and 76 in *FKS2* [45]. Of the three echinocandins, caspofungin seemed to be the most sensitive indicator of *FKS* mutations, whereas only four breakthrough isolates did not develop an *FKS* hot spot mutation despite showing greater than four-fold increases in echinocandin MICs relative to the parental isolates [45]. Of note, the rates of spontaneous *FKS* mutations observed with caspofungin were higher than with anidulafungin or micafungin [45]. Therefore, in our case, the use of caspofungin as a strong inducer of *FKS* mutations may have resulted in the rapid development of echinocandin resistance and subsequent therapeutic failure.

Although non-*FKS*-mediated echinocandin resistance has been reported [46,47], phenotypic resistance (MICs above CLSI breakpoints) to all three echinocandins is uniquely attributable to the presence of mutations in hot spots of both *FKS1* and its paralog *FKS2* [48], which results in attenuated echinocandin activity [49]. As recommended by the current Infectious Diseases Society of America (IDSA) guidelines [37], we performed echinocandin susceptibility testing on the *C. glabrata* isolates causing candidemia in our patient. Thus, we documented that isolate 2 (“breakthrough” isolate), compared to isolate 1 (“parental” isolate), had increased MIC values of anidulafungin, caspofungin and micafungin, and all values were higher than the CLSI resistance breakpoints [22]. As specifically shown for *C. glabrata* and echinocandins [50], the automated blood culture systems currently used to detect bloodstream infections allow for the reliable recovery of isolate populations composed of echinocandin-resistant and echinocandin-susceptible cells. However, in cases with a low proportion of resistant cells, picking up single colonies to perform standard antifungal susceptibility testing may result in missed detections of echinocandin resistance [50]. In our case, taking advantage of morphologically different *C. glabrata* colonies [51] from the patient’s blood culture that yielded isolate 2, we were able to detect echinocandin resistance by testing more than one colony. Consistent with recent studies [16,26], we found that isolate 2 harbored the *FKS2* HS1 F659Y. In a two-year antifungal resistance surveillance study [16], 8 (15.7%) of 51 *C. glabrata* isolates with *FKS* HS alterations harbored the *FKS2* HS1 F659S/V/Y [25,52], which was the second found after the *FKS2* HS1 S663P (16 isolates). It is noteworthy that mutations at positions S663 and F659 tended to be associated with breakthrough infections in patients receiving echinocandin therapy [25,53]. In our case, the MIC results (later confirmed by *FKS* mutation results) were promptly available to clinicians, but given the patient’s critical condition, the ensuing change of antifungal therapy was unsuccessful. Nevertheless, we acknowledge the importance of combining both antifungal susceptibility testing and *FKS* sequencing to predict therapeutic failure in candidemia patients treated with echinocandins [15]. This combination strategy would allow for encompassing cases of mutations occurring outside of HS *FKS* regions in echinocandin-resistant isolates [54], or cases of echinocandin-susceptible isolates carrying mutations in HS *FKS* regions in which the patients infected with such isolates show therapeutic failure following echinocandin treatment [55]. Ultimately, this strategy would ensure the choosing of an appropriate antifungal therapy in the clinic [15].

In conclusion, this case highlights that bacterial and fungal coinfections, including those associated with antimicrobial resistance, in COVID-19 may be a further challenge for both clinicians and microbiologists. In waiting for epidemiological studies to evaluate their frequency and impact, it is imperative to be vigilant for these coinfections when contemplating the outcome of COVID-19.

## Figures and Tables

**Table 1:** Antifungal susceptibility testing and *FKS2* gene sequencing results of two sequential candidemia isolates.

Species	Isolate	MIC (mg/L) for Polyene Antifungal Class	MIC (mg/L) for Echinocandin Antifungal Class			MIC (mg/L) for Azole Antifungal Class				*FKS2* Gene Hot Spots 1 and 2	
		AMB	AFG	CAS	MFG	FLZ	ITC	POS	VRC	Nucleotide Change	Amino acid Change
*C. glabrata*	Isolate 1	0.5	0.03	0.06	0.03	8	0.5	1	0.25	Wild type	Wild type
*C. glabrata*	Isolate 2	0.5	2	8	8	8	0.5	1	0.25	T1976A	F659Y
										A3997T	I1333F
										C4002T	A1334A (wild type)

Abbreviations: MIC, minimum inhibitory concentration; AMB, amphotericin B; AFG, anidulafungin; CAS, caspofungin; MFG, micafungin; FLZ, fluconazole; ITC, itraconazole; POS, posaconazole; VRC, voriconazole. Antifungal-resistant breakpoint values established by the CLSI for *C. glabrata* are ≥0.5 mg/L for anidulafungin and caspofungin, ≥0.25 mg/L for micafungin, and ≥64 mg/L for fluconazole. Because no resistance breakpoints were available for other listed antifungal agents, we used epidemiological cutoff values (ECVs) established by the CLSI for *C. glabrata*, according to which the non-wild-type MIC values (>ECVs) of amphotericin B, itraconazole, posaconazole and voriconazole are >2 mg/L, >4 mg/L, >1 mg/L and >0.25 mg/L, respectively.
